# The health-related quality of life in patients with post-COVID-19 after hospitalization: a systematic review

**DOI:** 10.1590/0037-8682-0741-2021

**Published:** 2022-03-28

**Authors:** Eduardo Augusto Barbosa Figueiredo, Whesley Tanor Silva, Sabrina Pinheiro Tsopanoglou, Débora Fernandes de Melo Vitorino, Luciano Fonseca Lemos de Oliveira, Keity Lamary Souza Silva, Hiago Daniel Herédia Luz, Matheus Ribeiro Ávila, Lucas Fróis Fernandes de Oliveira, Ana Cristina Rodrigues Lacerda, Vanessa Amaral Mendonça, Vanessa Pereira Lima, Mauro Felippe Felix Mediano, Pedro Henrique Scheidt Figueiredo, Manoel Otávio Costa Rocha, Henrique Silveira Costa

**Affiliations:** 1 Universidade Federal dos Vales do Jequitinhonha e Mucuri, Residência em Fisioterapia na Saúde Coletiva, Diamantina, MG, Brasil.; 2 Universidade Federal dos Vales do Jequitinhonha e Mucuri, Programa de Pós-Graduação em Reabilitação e Desempenho Funcional, Diamantina, MG, Brasil.; 3 Universidade Federal dos Vales do Jequitinhonha e Mucuri, Departamento de Fisioterapia, Diamantina, MG, Brasil.; 4 Universidade Federal de Minas Gerais, Escola de Educação Física, Fisioterapia e Terapia Ocupacional, Belo Horizonte, MG, Brasil.; 5 Fundação Oswaldo Cruz, Instituto Nacional de Infectologia Evandro Chagas, Rio de Janeiro, RJ, Brasil.; 6 Universidade Federal de Minas Gerais, Curso de Pós-Graduação em Infectologia e Medicina Tropical, Belo Horizonte, MG, Brasil.

**Keywords:** COVID-19, SARS-CoV-2, Hospitalization, Quality of life

## Abstract

Symptoms in post-COVID-19 patients who require hospitalization can persist for months, significantly affecting their health-related quality of life (HRQoL). Thus, the present study aimed to discuss the main findings regarding HRQoL in post-COVID-19 patients who required hospitalization. An electronic search was performed in the MEDLINE, EMBASE, CINAHL, Web of Science, LILACS, and Scopus databases, without date and language restrictions, until July 2021. Twenty-four articles were included in the analysis. It seems that HRQoL partially improved soon after hospital discharge, although the negative impact on HRQoL may persist for months. The physical and mental aspects are affected because patients report pain, discomfort, anxiety, and depression. The HRQoL of COVID-19 infected patients was worse than that of uninfected patients. Additionally, HRQoL seemed worse in patients admitted to the intensive care unit than in those who remained in the ward. Improvements in HRQoL after hospital discharge are independent of imaging improvement, and there seems to be no association between HRQoL after hospital discharge and disease severity on hospital admission. Many factors have been identified as determinants of HRQoL, with women and advanced age being the most related to worse HRQOL, followed by the duration of invasive mechanical ventilation and the need for intensive care. Other factors included the presence and number of comorbidities, lower forced vital capacity, high body mass index, smoking history, undergraduate education, and unemployment. In conclusion, these findings may aid in clinical management and should be considered in the aftercare of patients.

## INTRODUCTION

COVID-19 is an acute respiratory infection caused by the potentially serious SARS-CoV-2, with high transmissibility and global distribution^1^. SARS-CoV-2 is a beta-coronavirus that was discovered in the city of Wuhan, China, in December 2019. Coronaviruses are a large family of viruses common to different species of animals^2^. 

COVID-19 has generated great concern in the population due to its ability to cause serious conditions in a large proportion of infected patients^3^ ^,^ ^4^. Approximately 20% of hospitalized patients develop severe complications, including respiratory failure, acute respiratory distress syndrome (ARDS), shock, delirium, and multiple organ dysfunction^5^ ^,^ ^6^. In addition, critical patients greatly require therapies such as mechanical ventilation, which usually requires prolonged intensive care unit stays and post-COVID-19 rehabilitation^7^ ^,^ ^8^. Thus, such factors can decrease health-related quality of life (HRQoL) due to the physical, cognitive, and mental impairments of individuals with critical illnesses^9^ ^,^ ^10^.

HRQoL provides a complete assessment of the impact of a disease on patients' daily lives^11^. A structured review^12^ was recently conducted to verify the scores from different HRQoL questionnaires in post-COVID-19 patients. However, the general aspects of HRQoL after hospital discharge still require discussion. Even with no need for hospitalization, many patients may have a worse HRQoL than non-infected individuals^13^. However, due to the prolonged length of hospital stay, the need for invasive mechanical ventilation, pain, and fear of death, the investigation of HRQoL among hospitalized patients is of paramount importance. Therefore, the present study proposes systematically discussing the main findings of HRQoL in patients post-COVID-19 that required hospitalization. Establishing the general aspects of the HRQoL of these patients and identifying their determinants can help in the management of patients after hospital discharge.

## METHODS

### Study design

This systematic review aimed to discuss the main findings on the HRQoL of post-COVID-19 patients after hospitalization. The study was edited following the PRISMA checklist^14^ and Cochrane recommendations^15^. The protocol was prospectively registered in the open science framework (https://osf.io/k9pu6/).

### Search strategy and study selection

Search strategies were conducted using the Medical Literature Analysis and Retrieval System Online (MEDLINE), EMBASE, the Cumulative Index to Nursing and Allied Health Literature (CINAHL), Web of Science, Latin American and Caribbean Health Sciences Literature (LILACS), and Scopus databases. There were no language restrictions from their inception until July 2021. The following strategy was be used for the PubMed search - [("COVID-19" OR "SARS-CoV-2" OR "post-acute COVID-19 syndrome" OR "SARS-CoV-2 variants" OR "COVID-19 post-intensive care syndrome" OR "COVID-19 stress syndrome") AND ("Quality of Life" OR "Life Quality" OR "Health-Related Quality Of Life" OR "Health Related Quality Of Life" OR "HRQOL")], being modified for each database. After the searches, the retrieved references were exported to an Endnote® file, removing duplicates. Two independent reviewers checked for full texts using titles and abstracts. Studies that met our eligibility criteria were included in the review, and discrepancies between reviewers were resolved by a third reviewer.

### Eligibility criteria

This review included published observational studies, such as cross-sections, cohort, or control case studies, which assessed HRQoL in post-COVID-19 patients, after hospitalization, from both sexes, and at any age. Eligibility criteria included studies a) that evaluated patients post-COVID-19 after hospitalization; b) and assessed HRQoL. The exclusion criteria were articles in duplicate, studies that did not report the questionnaire used, papers that aimed to verify the improvement of HRQoL after rehabilitation, and those that did not match the objective of this review. In addition, studies that assessed only the questionnaire’s psychometric properties were also excluded, as it is not yet possible to establish a gold standard.

### Quality assessment

Two independent reviewers (EABF and WTS) assessed the methodological quality of the included studies using the Newcastle-Ottawa Scale^16^. This scale assigns a maximum of ten points for the lowest risk of bias and zero for the highest risk of bias. Newcastle-Ottawa scores the risk of study bias in three domains: 1) selection of study groups (four points), 2) group comparability (two points), and 3) verification of exposure and results (three points) for case-control and cohort studies, respectively. 

For cross-sectional studies, Newcastle-Ottawa Scale has a maximum score of ten stars, divided into the topics "selection,” "comparability," and "results"^17^. To assess the risk of bias, studies that scored in all domains (selection, comparability, and outcome) were classified as high quality^18^. Those who did not score in at least one of the domains were classified as low-quality. All studies found in the electronic search, regardless of the methodological quality, were included in the review.

### Outcome and data analysis

The extracted data included the year of publication, sample characteristics, HRQoL questionnaire used, and results. We did not perform a meta-analysis, and the results were presented as descriptive data. The corresponding author was contacted in the presence of any missing data.

## RESULTS OF THE ELECTRONIC SEARCH

**FIGURE 1:**
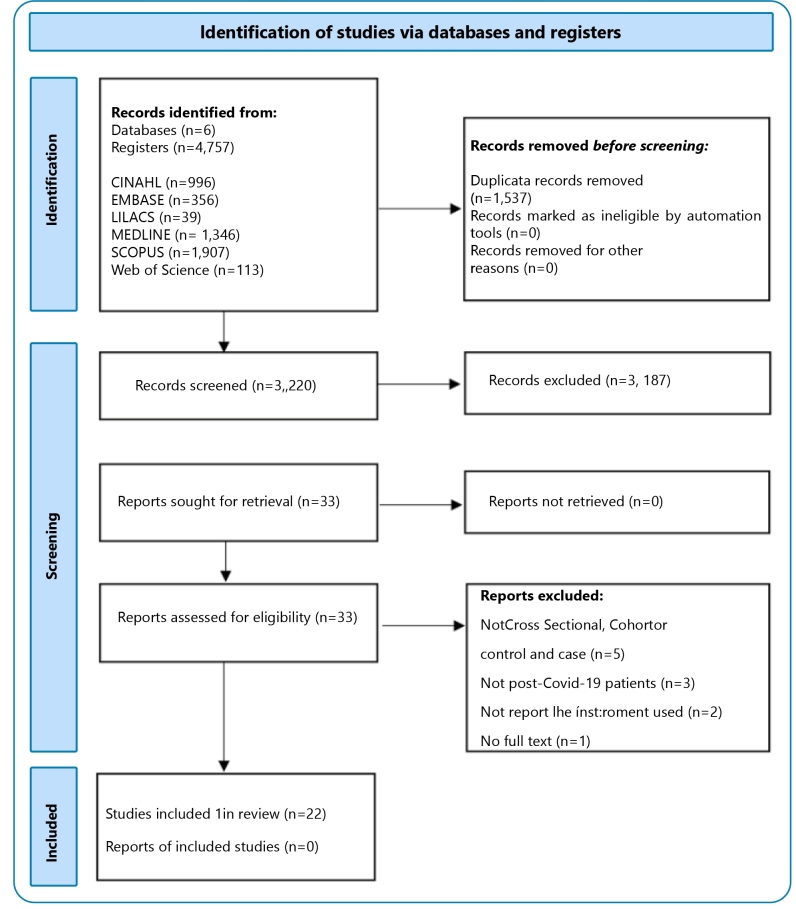
Flow diagram of studies through the review.

We retrieved 4757 titles, of which 1513 were duplicates and excluded. The remaining 3244 studies were screened, and 57 references were selected for inclusion in the study. Of these, 24 met our inclusion criteria. Figure 1 outlines the flow of the review papers.

Seven questionnaires were used in the included studies: generic (Short-form Health Survey [SF-36]/Rand-36, 12-item Short Form [SF-12]), Euro Quality of Life 5 dimensions 5 levels [EQ-5D-5L], Euro Quality of Life 5 dimensions 3 levels [EQ-5D-3L], World Health Organization Quality of Life [WHOQOL-Bref], 15D], and airways disease-specific [St George's Respiratory Questionnaire (SGRQ)]. 

The SF-36/Rand-36 is a generic questionnaire that encompasses 36 items grouped into eight domains: physical functioning, physical, bodily pain, general health, social functioning, role-emotional, vitality, and mental health. The total score ranges from 0 (lowest HRQL) to 100 (highest HRQL), and the higher the score, the better the patient's HRQoL^19^ ^,^ ^20^. The SF-12 is a generic HRQoL assessment instrument composed of a 12-item subset of SF-36. It assesses the same eight HRQoL domains of SF-36^21^ ^,^ ^22^.

The 5-level EQ-5D (EQ-5D-5L) has two parts: the EQ-5D descriptive system and the EQ visual analog scale (EQ-VAS). The descriptive system had five dimensions: mobility, self-care, usual activities, pain or discomfort, and anxiety or depression. In addition, the questionnaire allowed five levels of response: no problems, slight problems, moderate problems, severe problems, and extreme problems. Higher values indicate better health. The EQ-VAS records the patient's self-rated health on a vertical visual analogue scale, where the endpoints are labeled from “The best health you can imagine” to “The worst health you can imagine”^23^. Another version, EQ-5D-3L, is similar to the EQ-5D-5L. However, it has three response levels: no problems, some problems, and extreme problems.

The WHOQOL-Bref is a general instrument comprising 26 questions. The first question referred to the general HRQoL, the second to self-satisfaction, and the other 24 to the physical, psychological, social relations, and environment domains^24^. The higher the score, the better the HRQoL of the patient.

The 15D is a generalist instrument that covers 15 dimensions: breathing, mental function, speech (communication), vision, mobility, usual activities, vitality, hearing, eating, elimination, sleeping, distress, discomfort and symptoms, sexual activity, and depression. It is a questionnaire designed to be administered within 10 minutes^25^. Higher scores indicate worse HRQoL.

Saint George's Respiratory Questionnaire (SGRQ) is one of the leading HRQoL assessment questionnaires specific to respiratory diseases. It comprises items divided into three sessions: symptoms, activity, and impacts (which cover a range of aspects concerned with social and psychological functioning). The score for each session and the total score can be calculated^26^. The higher the score, the worse is the HRQoL.

## GENERAL ASPECTS OF THE HRQOL OF POST-COVID-19 PATIENTS

Eleven studies (Table 1) verified the general aspects of HRQoL in patients post-COVID-19 after hospitalization^27^ ^-^ ^36^. The mean score for the quality of studies was 6 (range, 5-8). Six studies were classified as low-quality, while five were classified as high-quality. The questionnaires used were EQ-5D-5L, EQ-5D-3L, SGRQ, SF-12, WHOQOL-Bref, and SF-36. 

**TABLE 1:** General aspects of HRQoL in post COVID-19 patients (n=11).

Study	Sample characteristics	Objective related to HRQoL	Instrument	Follow-up	Outcomes	Methodological quality	Overall quality
Daher *et al*., 2020	n=33 patients after severe COVID-19 (mean 64.0 years, SD 3.0, 67% males; mean length of stay 15 days, SD 1.8)	To verify the HRQoL of patients after severe COVID-19 infection and to compare it with the HRQoL at the time of hospital admission.	EQ-5D-5L and SGRQ	6 weeks after hospital discharge	At the end of follow-up period, patients reported slight to moderate abnormalities in mobility, self-care, usual activities, pain/discomfort and anxiety/depression by EQ-5D-5L.	Selection (★★) Comparability (-) Exposure (★★★) **Total score:** 5/9	Low quality
Maheshwari *et al*., 2021	n=51 convalescent plasma donors who recovered from COVID-19 and were symptom free and negative (mean 34.37 years, SD 9.08, 90.2% males).	To analyze the psychological impact of COVID-19 among convalescent recovered plasma donors.	WHOQOL-Bref	Not reported	The worst affected WHOQOL-Bref domain was the physical, followed by environmental, psychological, and social relationships.	Selection (★★) Comparability (-) Exposure (★★★) **Total score**: 5/9	Low quality
Méndez *et al*., 2021	n=179 patients who were hospitalized with COVID-19 (ages ranging from 22 to 81 years, 41.3% females)	To assess the HRQoL and psychiatric symptoms in post-COVID-19 survivors after hospital discharge.	SF-12	2 months after hospital discharge	Low HRQoL for physical and mental components was detected in 44.1% and 39.1% of patients, respectively.	Selection (★★★★) Comparability (-) Exposure (★★) **Total score**: 6/9	Low quality
Monti *et al*., 2021	n=39 patients after intensive care unit discharge [mean 56 years, SD 10.5, 10.0% females, after mechanical ventilation for a median of 9 (6-14) days].	To assess the HRQoL of survivors of Severe Acute Respiratory Syndrome by COVID-19 ventilated invasively.	EQ-5D-5L	2 months [median 61 days (Q1-Q3: 51 to 71 days)] after intensive care unit discharge.	Patients showed no difficulty in walking (82%), self-care (85%) and usual activities (78%). Only eight (21%) patients reported anxiety or moderate depression.	Selection (★★) Comparability (-) Exposure (★★★) **Total score**: 5/9	Low quality
Navarro *et al*., 2020	n=115 patients after mild or moderate COVID-19 [median age of 40 years (Q1-Q3: 33 to 48 years), 57.0% females, 4% admitted at intensive care unit].	To identify the changes in HRQoL in the early convalescence phase of a group of recovered COVID-19 patients.	EQ-5D-5L	1 month after the onset of symptoms.	There was a severe decrease in HRQoL in 56% of patients. Abnormalities in usual activities and anxiety/depression were detected in 59% of patients with a severe decrease in HRQoL	Selection (★★) Comparability (-) Exposure (★★★) **Total score**: 5/9	Low quality
Qu *et al*., 2021	n=540 patients with post-COVID-19, in a multicenter Chinese study, [median age of 47.50 years (Q1-Q3: 37.0 to 57 years), 50.0% females, 90.4% with mild to moderate severity].	To compare the HRQoL of patients with COVID-19 after hospital discharge with general Chinese population and to verify the determinants of HRQoL after COVID-19.	SF-36	3 months after hospital discharge.	In the post-COVID-19 patients, 15.4% had poor physical component summary, and 32.6% had poor mental component summary.	Selection (★★★) Comparability (★★) Exposure (★★★) **Total score**: 8/9	High quality
Rass *et al*., 2021	n=135 patients with post-COVID-19 [(median age of 56 days (Q1-Q3: 48 to 68 days), 61.0% males, median length of stay of 8 days (Q1-Q3: 2 to 18 days)]	To identify the impact of COVID-19 on mental health and HRQoL 3 months after disease onset.	SF-36	3 months after disease onset.	The HRQoL was impaired in 31% of patients, and symptoms of depression, anxiety, and posttraumatic stress disorders were detected in 11%, 25%, and 11% of patients, respectively.	Selection (★★) Comparability (★) Exposure (★★★) **Total score**: 6/9	High quality
Santus *et al*., 2020	n=20 hospitalized patients after COVID-19-related pneumonia (mean 55 years, SD 15, 85% males, mean length of stay 17.7 days, SD 11.5).	To assess the HRQoL of patients with post-COVID-19-related pneumonia after hospitalization.	SGRQ	15 days after hospital discharge.	After 15 days of hospital discharge, there was significant improvement in the score of all domains of SGRQ, i.e., symptoms (mean 33.7, SD 18.0 *versus* 16.7, SD 12.9), activity (mean 35.7, SD 24.2 *versus* 28.3, SD 23.3), impact (mean 17.3, SD 15.9 *versus* 10.6, SD 10.7), and total score (mean 25.5, SD 15.5 *versus* 16.9, SD 13.2) (p<0.01 for all).	Selection (★★) Comparability (-) Exposure (★★★) **Total score**: 5/9	Low quality
Temperoni *et al*., 2021	n=64 patients diagnosed with COVID-19, and aged ≤50 years (mean 41.1 years, SD 7.4, 53.8% males, 31.7% hospitalized).	To report the HRQoL of patients after COVID-19 and aged ≤50 years.	SF-36	1 month after hospital discharge.	There were no significant differences between hospitalized and non-hospitalized patients in physical or mental component summaries. The mean of SF-36 physical component summary in the hospitalized and non-hospitalized patients were, respectively, 56.25, SD 23.15 *versus* 55.32, SD 23.48 (p=0.894); The mean of SF-36 mental component summary in the hospitalized and non-hospitalized patients were, respectively, 53.63, SD 28.11 *versus* 48.72, SD 23.14 (p=0.498)	Selection (★★) Comparability (★★) Exposure (★★★) **Total score**: 7/9	High quality
Todt *et al*., 2021	n=251 patients [mean 53.6, SD 14.9 years, 59.8% males, 69.7% with severe COVID-19 at admission, 13.6% at invasive mechanical ventilation, median length of stay of 5 days (Q1-Q3: 3 to 10 days)].	To assess the impact of COVID-19 on HRQoL, anxiety, and depression after hospital discharge and to verify the determinants of the worsening in HRQoL.	EQ-5D-3L	3 months after hospital discharge	Eighty one patients had a positive screening for anxiety/depression. The EQ-5D-3L index was reduced 3 months after discharge (median score 0.80) when compared to the onset of COVID-19 symptoms (median score 1.0) (p<0.001).	Selection (★★) Comparability (★★)Exposure (★★★) **Total score**: 7/9	High quality
Walle-Hansen *et al*., 2021	n=106 participants were hospitalized for COVID-19 (mean age 74.3 years and 56.6% males, 26% after severe COVID-19).	To compare the HRQoL before and after COVID-19.	EQ-5D-5L	6 months after hospitalization	Seventy patients reported a negative change in any of the dimensions of the EQ 5D-5L when compared to before COVID-19.	Selection (★★) Comparability (★★)Exposure (★★★) **Total score**: 7/9	High quality

**Abbreviations:** HRQoL: health-related quality of life; EQ-5D-5L: Euro Quality of life (5 dimensions and 5 levels); EQ-5D-3L: Euro Quality of life (5 dimensions and 3 levels); SGRQ: St George's Respiratory Questionnaire; WHOQOL-Bref: World Health Organization Quality of Life; SF-12: 12-items Short-form Health Survey; SF-36: 36-items Short-Form Health Survey; SD: standard deviation; Q1-Q3: interquartile range.

One study^34^ verified the changes in the HRQoL of post-Covid patients 15 days after hospital discharge. The authors demonstrated that 15 days were sufficient to detect significant improvements in all domains of the SGRQ (symptoms, activity, impact, and total score). However, COVID-19 symptoms commonly persist for 35 days, affecting both physical and mental health^37^. Despite the improvement after hospital discharge, Mendéz *et al*.^29^ demonstrated by the SF-12 that both the physical and mental component summaries were impaired in approximately 44.1% and 39.1% of patients, respectively, two months after hospital discharge. Even in patients with mild to moderate severity, impaired physical and mental component summaries were detected in 15.4% and 32.6% of the patients, respectively^32^. Therefore, the HRQoL of post-COVID-19 patients may remain worse during the months following discharge.

Temperoni *et al*.^38^ verified the HRQoL of 64 patients aged less than 50 years post-COVID-19 and showed no difference between hospitalized and non-hospitalized patients in any SF-36 domains after one month of hospital discharge. In a small sample (n=39), Monti *et al*.^30^ reported that, after two months of intensive care unit discharge, a majority of patients showed no difficulty in walking, self-care, and usual activities in EQ-5D-5L, and only eight patients reported anxiety or moderate depression. Using the same questionnaire, with a similar follow-up period (six weeks after hospital discharge) and a small sample, Daher *et al*.^27^ showed that patients reported only slight to moderate abnormalities with mobility, self-care, usual activities, pain/discomfort, and anxiety/depression. In contrast, three other studies found different results with larger samples and using the same questionnaire. Navarro *et al*.^31^ found that after mild to moderate COVID-19 infection, a severe decrease in HRQoL was observed in 56% of patients, mainly in usual activities and anxiety/depression. Another study^35^ found that 32% of patients had anxiety/depression and 38% reported worsening HRQoL three months after hospital discharge (p<0.001), especially in pain/discomfort and anxiety/depression. Finally, Walle-Hansen^36^ showed that 66% of patients (n=106) reported a negative change in any of the dimensions of the EQ 5D-5L when compared to the time before COVID-19. These changes are related to difficulties in performing activities of daily living, reduced mobility, and pain or discomfort. In summary, according to studies with larger samples, pain/discomfort (physical aspects) and anxiety/depression (mental aspects) were the most compromised domains in post-COVID-19 patients after hospitalization.

More studies in post-COVID-19 patients are needed, but patient-reported pain may have multiple causes. Pain may be a consequence of a viral infection in the peripheral neuromuscular or central nervous system, or may occur as a result of invasive mechanical ventilation, or may be secondary to associated syndromes, such as Guillain-Barré syndrome^39^. Joint pain, chest pain, headache, and myalgia are among the most cited symptoms by patients^40^ ^,^ ^41^.

Regarding the mental aspects, according to Mendéz *et al*.^29^, anxiety, depression, and post-traumatic stress disorder were detected in 29.6%, 26.8%, and 25.1% of patients, respectively. In addition, 39.1% of patients had psychiatric impairments. Another study^33^ demonstrated that HRQoL was impaired in 31% of patients, and symptoms of depression, anxiety, and post-traumatic stress disorders were detected in 11%, 25%, and 11% of the patients, respectively. Maheshwari *et al*.^28^ also found that fear of reinfection by hospital exposure and social stigma was experienced by 51% and 49% of plasma donors, respectively. These results suggest that not only the physical aspects but also the mental health, anxiety, depression, and fear of post-COVID-19 patients should be addressed in post-discharge follow-up.

Thus, the results suggest that (1) some improvements in HRQoL are detectable a few days after hospital discharge, (2) poor HRQoL may persist for months after discharge, and (3) impairments in both physical (pain and discomfort) and mental (anxiety and depression) aspects are present and must be addressed in patient management.

## COMPARISON BETWEEN HRQOL BETWEEN PATIENTS WITH POST-COVID-19 AND UNINFECTED POPULATION.

Six included studies^32^ ^,^ ^42^ ^-^ ^46^ compared the HRQoL between post-Covid patients and uninfected individuals (Table 2). The questionnaires used were SF-36, 15D, SF-12, and SGRQ. The mean quality score was 7 (range, 6-8). Five were classified as high-quality, while only one was of low quality.

**TABLE 2:** HRQoL between post-COVID-19 patients and the general population (n=6).

Study	Sample characteristics	Objective related to HRQoL	Instrument	Follow-up	Outcomes	Methodological quality	Overall quality
Chen *et al*., 2020	n=361 post-COVID-19 patients one month after hospital discharge (mean 47.22 years, SD 13.03; 51.5% males; 9.4% severe cases; mean length of stay of 19.13 days, SD 7.60 days).	To verify the difference in HRQoL between post-COVID-19 patients one month after hospital discharge and healthy Chinese individuals and to identify the determinants of HRQoL in post-COVID-19 patients	SF-36	1 month after hospital discharge	All SF-36 domains were reduced in post-COVID-19 patients when compared to the healthy Chinese population, with the exception of the score in the physical functioning domain. In the physical functioning domain, there was no difference between male post-COVID-19 patients and male Chinese general population (mean 95.13, SD 9.11 *versus* 95.60, SD 10.43, respectively; p=0.43), and between female post-COVID-19 patients and female Chinese general population (mean 93.17, SD 10.26 *versus* 92.57, SD 13.88, respectively; p=0.41).	Selection (★★★) Comparability (★) Outcome (★★) **Total score:** 6/10*	High quality
Gamberini *et al*,. 2021	n=205 patients with post-COVID-19 after intensive care unit discharge [( median age of 63 years (55 to 70 years), 74.1% males), median length of hospital stay was 42 days (Q1-Q3: 31 to 57 days).	To evaluate the HRQoL at 90 days after intensive care unit discharge and to verify the factors related to HRQoL.	15D	3 months after intensive care unit discharge	The 15D score was significantly lower in patients admitted at intensive care unit (mean 0.850, SD 0.143) than the two matched controls from Italian (mean 0.929, SD 0.809) and Finnish (mean 0.914, SD 0.084) samples of the general population (p<0.001 for both).	Selection (★★★) Comparability (★) Exposure (★★★) **Total score**: 7/9	High quality
Gianella *et al*., 2021	n=39 consecutive patients with post-COVID-19-related pneumonia [median 62.5 (Q1-Q3: 51.3 to 71.0 years; 76.9% males; median length of stay 15.0 (Q1-Q3:12.0 to 22.0 days)]	To verify the HRQoL after 3 months of follow-up and compare the HRQoL between groups with and without improvements on chest computed tomography.	SF-12 and SGRQ	3 months after hospital admission	The score evaluated by both SGRQ and SF-12 was significantly worse in post-Covid-related pneumonia patients (mean 16.97 and 30.97, respectively) when compared to the general population (reference values are 6 and 50, respectively) (p<0.0001).	Selection (★★★) Comparability (-) Exposure (★★★) **Total score**: 6/9	Low quality
Qu *et al*., 2021	n=540 patients with post-COVID-19, in a multicenter Chinese study, [median age of 47.50 years (Q1-Q3: 37.0 to 57 years), 50.0% females, 90.4% with mild to moderate severity].	To compare the HRQoL of patients with COVID-19 after hospital discharge with general Chinese population and to verify the determinants of HRQoL after COVID-19.	SF-36	3 months after hospital discharge.	The mean of SF-36 scores in the post-COVID-19 patients and in the Chinese population were, respectively: Physical functioning: 87.17, SD 14.57 *versus* 94.02, SD 12.44 (p<0.001); Role physical: 66.30, SD 41.04 *versus* 88.79, SD 28.49 (p<0.001); Bodily pain: 79.48, SD 20.73 *versus* 88.18, SD 19.02 (p<0.001); General heath: 68.90, SD 22.16 *versus* 69.74, SD 20.95 (p=0.393); Vitality: 55.35, SD 14.58 *versus* 68.92, SD 18.78 (p<0.001); Social functioning: 66.41, SD 24.51 *versus* 88.03, SD 16.00 (p<0.001); Role emotional: 71.30, SD 38.70 *versus* 89.57, SD 27.95 (p<0.001); Mental health: 22.86, SD 14.00 *versus* 77.61, SD 15.85 (p<0.001)	Selection (★★★) Comparability (★★) Exposure (★★★) **Total score**: 8/9	High quality
Raman *et al*., 2021	n=58 patients with post-COVID-19 [mean 55 years, SD 13, 59% males, median length of stay of 8.5 days (Q1-Q3: 5.0 to 17.0 days)] and 30 uninfected individuals matched for age, sex, body mass index and risk factors (smoking, diabetes and hypertension) from the community (during the same period).	To compare the HRQoL of post-COVID-19 patients with uninfected individuals.	SF-36	From 2 to 3 months from disease-onset.	The median of SF-36 scores in the post-COVID-19 patients and in uninfected individuals were, respectively: Physical functioning: 65.0, Q1-Q3: 45.0 to 90.0 *versus* 92.5, Q1-Q3: 83.8 to 100.0 (p<0.001); Role physical: 25.0, Q1-Q3: 0.0 to 75.0 *versus* 100.0, Q1-Q3: 100.0 to 100.0 (p<0.001); Role emotional: 33.3, Q1-Q3: 0.0 to 100.0 *versus* 100.0, Q1-Q3: 100.0 to 100.0 (p<0.001); Vitality: 45.0, Q1-Q3: 25.0 to 70.0 *versus* 65.0, Q1-Q3: 55.0 to 80.0 (p<0.001); Mental health: 76.0, Q1-Q3: 62.0 to 88.0 *versus* 84.0, Q1-Q3: 72.0 to 92.9 (p=0.044); Social functioning: 50.0, Q1-Q3: 37.5 to 87.5 *versus* 100.0, Q1-Q3: 62.5 to 100.0 (p<0.001); Bodily pain: 67.5, Q1-Q3: 35.0 to 90.0 *versus* 85.0, Q1-Q3: 67.5 to 100.0 (p=0.003); General heath: 68.8, Q1-Q3: 43.8 to 81.3 *versus* 75.0, Q1-Q3: 60.9 to 87.5 (p=0.022).	Selection (★★★) Comparability (★★) Exposure (★★★) **Total score**: 8/9	High quality
van der Sar-van der Brugge *et al*., 2021	n=101 participants after COVID-19-related pneumonia (mean 66.4 years, SD 12.6, 57.4% males, 72.3% after severe pneumonia).	To compare the HRQoL of patients after COVID-19-related pneumonia with the general Dutch population.	SF-36	6 weeks after hospital discharge.	When compared to general Dutch population, impaired HRQoL was found in almost all domains of the SF-36, except for bodily pain. The domains with the greatest commitment were physical role limitation, physical functioning and vitality.	Selection (★★) Comparability (★★) Exposure (★★★) **Total score**: 7/9	High quality

**Abbreviations:** HRQoL: health-related quality of life; SF-36: 36-items Short-Form Health Survey; SF-12: 12-items Short-Form Health Survey; SGRQ: St George's Respiratory Questionnaire; SD: standard deviation; Q1-Q3: interquartile range. *NOS for cross-sectional studies can score up to 10 stars.

Two studies compared the HRQoL evaluated by SF-36 between patients post-COVID-19 and data about the general Chinese population. One study^42^ demonstrated that the HRQoL of post-COVID-19 patients was reduced in all SF-36 domains, except for physical functioning, one month after hospital discharge. Another one^32^ reported that the HRQoL of post-COVID-19 patients was worse in many domains, except for general health, after three months of hospital discharge. In the Dutch population, it was demonstrated that the HRQoL evaluated by SF-36 was reduced in many domains after six weeks of hospital discharge, except for bodily pain^46^.

In Italian and Finnish populations, Gamberini *et al*.^43^ demonstrated that the HRQoL assessed using the 15D instrument was also worse when compared to data from the general population. In a small sample, another study^44^ demonstrated that HRQoL was significantly reduced in patients with post-COVID-19-related pneumonia when compared to the general population three months after hospital admission.

Finally, Raman *et al*.^45^ selected 58 patients diagnosed with COVID-19 and assessed HRQoL using SF-36 from two to three months after symptom onset and compared the HRQoL with a control group (n=30 uninfected individuals matched for age, sex, body mass index, and risk factors). The authors demonstrated that HRQoL was worse in all the SF-36 domains. 

Briefly, all included data were consistent, with good methodological quality, and showed that the HRQoL of post-COVID-19 patients was worse than that of the uninfected group even after hospital discharge. These findings can be explained by factors such as pulmonary impairment^47^, fatigue, muscular pain^37^, and anxiety^31^.

### THE HRQOL OF POST-COVID-19 PATIENTS ADMITTED AND NOT ADMITTED TO THE INTENSIVE CARE UNIT

Five studies^38^ ^,^ ^41^ ^,^ ^48^ ^-^ ^51^ compared HRQoL between patients admitted and those not admitted to the intensive care unit (Table 3). The questionnaires used were WHOQOL-Bref and EQ-5D-5L. The mean quality score was 6.4 (ranging, 5-7), were classified as high-quality.

**TABLE 3:** Comparison between the HRQoL of post-COVID-19 patients admitted and not admitted into intensive care unit (n=5).

Study	Sample characteristics	Objective related to HRQoL	Instrument	Follow-up	Outcomes	Methodological quality	Overall quality
Albu *et al*., 2021	n=30 individuals with persistent symptoms and/or sequelae of COVID-19 (16 post-intensive care unit, median age of 54 years (Q1-Q3: 43.8 to 62.0 years), 61.2% males, median length of hospital stay of 37 days (Q1-Q3: 15 to 69 days).	The compare the HRQoL of post-COVID-19 patients that were admitted ate intensive care unit with those that who not and to verify the correlation between HRQoL and fatigue and anxiety/depression.	WHOQOL-Bref	>3 months after acute COVID-19	There were no differences between groups admitted at post-intensive care unit and without post-intensive care unit in any WHOQOL-Bref domain.	Selection (★★★) Comparability (★★) Outcome (★★) **Total score:** 7/10*	High quality
Halpin *et al*., 2020	n=100 patients post-COVID-19, divided into ward group [68 patients; median age of 70.5 years (Q1-Q3: 20 to 93 years), 51.5% males; median length at ward of 6.5 days (Q1-Q3: 4 to 14)] and ICU group [(32 patients; median age of 58.5 years; 59.4% males; median length at intensive care unit of 4 days (Q1-Q3: 2.6 to 5.7 days).	To identify the impact of COVID-19 on HRQoL of discharged survivors.	EQ-5D-5L	Between 4 to 8 weeks after discharge	There was a clinically significant drop in EQ5D by 68.8% in the intensive care unit group and 45.6% in the ward group.	Selection (★★★) Comparability (★) Outcome (★★) **Total score**: 5/10*	High quality
Garrigues *et al*., 2020	n=120 patients post-COVID-19 stratified into ward group [96 patients; mean 64.1 years, SD 16.1), 58.3% males; mean length of stay in hospital 7.4 days, SD 5.4, and intensive care unit group (24 patients; mean 59.6 years, SD 13.7; 79.2% males; mean length of stay in hospital 26.5 days, SD 22.3).	To verify the difference in HRQoL between patients admitted at ward and at intensive care unit.	EQ-5D-5L	Mean of 110.9 days after admission for COVID-19.	There was no difference in HRQoL between group admitted at ward *versus* intensive care unit both in EQ-5D-5L (mean score 0.86, SD 0.19 *versus* 0.82, SD 0.21, respectively; p=0.306), and Visual analogic scale (mean score 69.9, SD 21.4 *versus* 71.7, SD 22.2, respectively; p=0.711).	Selection (★★★) Comparability (★★) Outcome (★★) **Total score**: 7/10*	High quality
Huang *et al*., 2021	n=1733 patients after hospital discharge from COVID-19; median 57.0 years (Q1-Q3: 47.0 to 65.0 years; median length of stay of 14.0 (Q1-Q3: 10.0 to 19.0 days), 52% men. Patients were stratified into three groups: that did not require oxygen supplementation; that required oxygen supplementation; and that required high-flow nasal cannula, or non-invasive mechanical ventilation, or invasive mechanical ventilation.	To verify the difference in HRQoL among the three groups of patients.	EQ-5D-5L	Median 186.0 (Q1-Q3: 175.0 to 199.0) days.	When compared to patients that did not require supplemental oxygen, patients with high-flow nasal cannula, non-invasive mechanical ventilation, or invasive mechanical ventilation had more problems in mobility (6% *versus* 14%), pain or discomfort (26% *versus* 41%), and anxiety or depression (23% *versus* 32%).	Selection (★★) Comparability (★★) Exposure (★★★) **Total score**: 7/9	High quality
Lerum *et al*., 2021	n=103 patients post- COVID-19, stratified in group after intensive care unit [n=15, median age 52 years (Q1-Q3: 50 to 59 years; 73% males; median length of stay 17 days (Q1-Q3: 12 to 25 years)] and no intensive care unit [n=88; median age 61 years (Q1-Q3: 49 to 74 years; 49% males, median length of stay 5.0 (Q1-Q3: 3 to 9 days)]	To verify the difference in HRQoL between patients with and without admission at the intensive care unit.	EQ-5D-5L	3 months after hospital discharge	Patients admitted to the intensive care unit (median score 4, Q1-Q3: 2 to 4) had worse HRQoL in the domain usual activities than patients admitted only to regular wards (median score 2, Q1-Q3: 1 to 2, respectively) (p=0.014).	Selection (★★) Comparability (★) Exposure (★★★) **Total score**: 6/9	High quality

**Abbreviations:** HRQoL: health-related quality of life; WHOQOL-Bref: World Health Organization Quality of Life; EQ-5D-5L: Euro Quality of Life (five dimensions and five levels); SD: standard deviation; Q1-Q3: interquartile range. *NOS for cross-sectional studies can score up to 10 stars.

Halpin *et al*.^49^ were the first to verify the HRQoL of patients with post-COVID-19 admitted to the ward and in the intensive care unit. They demonstrated that in a short period, there was a significant improvement in HRQoL assessed by EQ-5D-5L in both groups. Using the same questionnaire, Garrigues *et al*.^41^ demonstrated no difference between groups of patients admitted to the ward and intensive care unit after 10 days after hospital discharge. Using the WHOQoL-Bref, Albu *et al*.^48^ also found no difference in HRQoL between patients with persistent symptoms admitted (n=16) and those not admitted (n=14) to the intensive care unit after three months of hospital discharge. Based on these results, it seems that being in the intensive care unit or adopting more invasive treatment strategies does not influence the HRQoL of patients after COVID-19. However, prolonged mechanical ventilation is associated with a more extended intensive care unit and hospital stays^52^, as well as significant and lasting physical and psychological dysfunction in critically ill survivors^53^.

Corroborating this sentence, two other studies demonstrated differences in HRQoL between patients admitted and not admitted to the intensive care unit, both using the EQ-5D-5L questionnaire. Huang *et al*.^50^ described, in a larger sample, that patients who needed supplemental oxygen, high-flow nasal cannula, non-invasive mechanical ventilation, or invasive mechanical ventilation had worse scores in the mobility, pain or discomfort, and anxiety or depression domains when compared to those without supplemental oxygen. Finally, Lerum *et al*.^51^ reported that the HRQoL of patients admitted to the intensive care unit was worse after prolonged hospital discharge (greater than three months) in the usual activities domain than that of patients admitted only to regular wards.

The results are conflicting, despite the high methodological quality. However, four studies, using regression analysis, demonstrated that the need for intensive care unit^35^ ^,^ ^54^ or duration of invasive mechanical ventilation^43^ ^,^ ^55^ were independent determinants of the HRQoL of post-COVID-19 patients. Thus, it seems that patients admitted to the intensive care unit have a worse HRQoL than those not admitted, even after hospital discharge.

### FACTORS ASSOCIATED AND DETERMINANTS OF HRQOL IN PATIENTS WITH POST-COVID-19

Identifying factors associated with the HRQoL of post-Covid patients is required to assist in patient stratification and guide clinical management. Nine included studies (Table 4) verified the factors associated with HRQoL in post-COVID-19^32^ ^,^ ^35^ ^,^ ^42^ ^-^ ^44^ ^,^ ^48^ ^,^ ^54^ ^-^ ^56^, with a mean score of 6.6 (ranging, 5-8). Seven of these were classified as high quality. The questionnaires used were WHOQOL-Bref, SF-12, EQ-5D-5L, SF-36, 15D, SGRQ, RAND-36, and EQ-5D-3L.

**TABLE 4:** Factors associated and determinants of HRQoL in patients with post-COVID-19 (n=9).

Study	Sample characteristics	Objective related to HRQoL	Instrument	Follow-up	Outcomes	Methodological quality	Overall quality
Albu *et al*., 2021	n=30 individuals with persistent symptoms and/or sequelae of COVID-19 (16 post-intensive care unit, median age of 54 years (Q1-Q3: 43.8 to 62.0 years), 61.2% males, median length of hospital stay of 37 days (Q1-Q3: 15 to 69 days).	The compare the HRQoL of post-COVID-19 patients that were admitted ate intensive care unit with those that who not and to verify the correlation between HRQoL and fatigue and anxiety/depression.	WHOQOL-Bref	>3 months after acute COVID-19	In general, HRQoL correlated with the impact of fatigue and anxiety/depression, except for the environment domain.	Selection (★★★) Comparability (★★) Outcome (★★) **Total score:** 7/10*	High quality
Anastasio *et al*., 2021	n=379 patients after the diagnosis of COVID-19, [median age of 56 years old (Q1-Q3: 49 to 63), 45.9% males, 222 after pneumonia, 161 without acute respiratory distress syndrome and 61 after acute respiratory distress syndrome].	To correlate the physical and mental component summaries of the SF-12 with lung function, development of pneumonia, acute respiratory distress syndrome, invasive mechanical ventilation, partial oxygen saturation/fraction of inspired oxygen ratio or pneumonia severity index.	SF-12	4 months after COVID-19 diagnosis	There was no significant correlation between physical or mental component summary with lung function, development of pneumonia, acute respiratory distress syndrome, invasive mechanical ventilation, partial oxygen saturation/fraction of inspired oxygen ratio or pneumonia severity index.	Selection (★★) Comparability (-) Exposure (★★★) **Total score**: 5/9	Low quality
Arab-Zozani *et al*., 2020	n=409 post-COVID-19 patients, mean 58.4 years, SD 18.21, 60.27% males, mean length of hospital stay 8 days, SD 7.	To verify the determinants of HRQoL	EQ-5D-5L	Mean 21.6 days, SD 14.8	Female sex, age> 50 years, university degree, be unemployed, presence of diabetes, diagnosis of heart failure, and admission to the intensive care unit were independent determinants of the HRQoL.	Selection (★★★) Comparability (★★) Outcome (★★) **Total score**: 7/10*	High quality
Chen *et al*., 2020	n=361 post-COVID-19 patients one month after hospital discharge (mean 47.22 years, SD 13.03; 51.5% males; 9.4% severe cases; mean length of stay of 19.13 days, SD 7.60).	To verify the difference in HRQoL between post-COVID-19 patients one month after hospital discharge and healthy Chinese individuals and to identify the determinants of HRQoL in post-COVID-19 patients	SF-36	1 month after hospital discharge	Factors associated HRQoL in post-COVID-19 patients were age, female sex, clinical subtype of the disease, chronic kidney disease, length of stay, smoking history and forced vital capacity. The determinants of lower physical component scores were overweight and obesity. The determinant of the mental component was female sex.	Selection (★★★) Comparability (★) Outcome (★★) **Total score**: 6/10*	High quality
Gamberini *et al*., 2021	n=205 patients with post-COVID-19 after intensive care unit discharge [(median age of 63 years (55 to 70 years), 74.1% males), median length of hospital stay was 42 days (Q1-Q3: 31 to 57 days).	To evaluate the HRQoL at 90 days after intensive care unit discharge and to verify the factors related to HRQoL.	15D	3 months after intensive care unit discharge	Age, female sex, number of comorbidities, acute respiratory distress syndrome class, duration of invasive mechanical ventilation, and occupational status were found to be significant determinants of the 90 days HRQoL. Clinical severity at admission was poorly correlated to HRQoL.	Selection (★★★) Comparability (★) Exposure (★★★) **Total score**: 7/9	High quality
Gianella *et al*., 2021	n=39 consecutive patients with post-COVID-19-related pneumonia [median 62.5 (Q1-Q3: 51.3 to 71.0 years; 76.9% males; median length of stay 15.0 (Q1-Q3:12.0 to 22.0 days)]	To verify the HRQoL after 3 months of follow-up and compare the HRQoL between groups with and without improvements on chest computed tomography.	SF-12 and SGRQ	3 months after hospital admission	After 3 months, there was no difference in SF-12 and SGRQ score between those who improved the chest computed tomography scan and those who did not (p>0.05). The median of SF-12 scores in the post-COVID-19 patients who improved the chest computed tomography scan and those who did not were, respectively: Physical domain: 53.1, Q1-Q3: 41.9 to 56.0 *versus* 32.5, Q1-Q3: 28.9 to 52.5 (p=0.051); and mental domain: 57.5, Q1-Q3: 49.0 to 59.9 *versus* 53.1, Q1-Q3: 41.4 to 58.9 (p=0.64). The median of SGRQ scores in the post-COVID-19 patients who improved the chest computed tomography scan and those who did not were, respectively, 9.9, Q1-Q3: 7.2 to 16.2 *versus* 20.4, Q1-Q3: 8.1 to 50.5 (p=0.16)	Selection (★★★) Comparability (-) Exposure (★★★) **Total score**: 6/9	Low quality
Lindahl *et al*., 2021	n=54 male patients (mean 60 years, SD 11, mean length of hospital stay 18 days, SD 17) and 47 female patients (mean 59 years, SD 11, mean length of hospital stay 12 days, SD 8)	To verify the determinants of HRQoL in patients with post-COVID-19 after hospitalization.	RAND-36	6 months	Age, female sex, BMI, sleep apnoea, and duration of mechanical ventilation were associated with worse HRQoL.	Selection (★★★) Comparability (★★) Outcome (★★) **Total score**: 7/10*	High quality
Qu *et al*., 2021	n=540 patients with post-COVID-19, in a multicenter Chinese study [median age of 47.50 years (Q1-Q3: 37.0 to 57 years), 50.0% females, 90.4% with mild to moderate severity].	To compare the HRQoL of patients with COVID-19 after hospital discharge with general Chinese population and to verify the determinants of HRQoL after COVID-19.	SF-36	3 months after hospital discharge.	Female sex, older age (≥ 60 years) and physical symptoms were associated with poor physical component summary; the physical symptom after discharge was associated with poor mental component summary.	Selection (★★★) Comparability (★★) Exposure (★★★) **Total score**: 8/9	High quality
Todt *et al*., 2021	n=251 patients [mean 53.6 years, SD 14.9, 59.8% males, 69.7% with severe COVID-19 at admission, 13.6% at invasive mechanical ventilation, median length of stay of 5 days (Q1-Q3: 3 to 10 days)].	To assess the impact of COVID-19 on HRQoL, anxiety, and depression after hospital discharge and to verify the determinants of the worsening in HRQoL.	EQ-5D-3L	3 months after hospital discharge	Only female sex and intensive care requirement were independently associated with worsening of HRQoL.	Selection (★★) Comparability (★★) Exposure (★★★) **Total score**: 7/9	High quality

**Abbreviations:** HRQoL: health-related quality of life; WHOQOL-Bref: World Health Organization Quality of Life; SF-12: 12-items Short-form Health Survey; SF-36: 36-items Short-Form Health Survey; SGRQ: St George's Respiratory Questionnaire; EQ-5D-3L: Euro Quality of Life (5 dimensions and 3 levels); SD: standard deviation; Q1-Q3: interquartile range.

One study^44^ reported no difference in SF-12 and SGRQ scores between groups who improved their chest computed tomography scan (n=31) and those who did not (n=8) after three months of hospital admission. Thus, there seems to be no association between imaging improvements and HRQoL after the follow-up period. Anastasio *et al*.^56^ also reported that there was no significant correlation between the physical or mental component summary of the SF-12 and lung function, development of pneumonia, acute respiratory distress syndrome, invasive mechanical ventilation, partial oxygen saturation/fraction of inspired oxygen ratio, or pneumonia severity index four months after COVID-19 diagnosis (n=379). Another study^43^ showed that clinical severity at admission was poorly correlated with HRQoL three months after intensive care unit discharge. According to these results, HRQOL was not associated with clinical measures. This can be explained by the fact that COVID-19 encompasses biological, psychological, and social factors, such as stigma and discrimination between various groups, as previously demonstrated^57^. However, many studies have reported fatigue and mental health issues in post-COVID-19 patients. Albu *et al*.^48^ showed a significant correlation between poor HRQoL, fatigue, and anxiety/depression.

Another six studies^32^ ^,^ ^35^ ^,^ ^42^ ^,^ ^43^ ^,^ ^54^ ^,^ ^55^ verified the independent determinants of HRQoL of patients in the post-COVID-19 period. The main determinants of the poor physical component of HRQoL were obesity and overweight^42^, female sex, older age (≥60 years), and the presence of physical symptoms after hospital discharge^32^. On the other hand, the independent determinants of poor mental components were women^42^ and the presence of physical symptoms after hospital discharge^32^. Thus, physical symptoms after hospital discharge and female sex were determinants of poor HRQoL in both physical and mental components in post-COVID-19 patients. 

In fact, in all six studies, female sex and old age were determinants of poor HRQoL in the post-COVID-19 period. It has been previously demonstrated that men have higher rates of disease severity and case-fatality^58^, but women suffer more from long-term symptoms than men^55^. Additionally, old age contributes to poor physical and mental health recovery status^32^.

Other determinants of poor HRQoL in post-COVID-19 patients include clinical subtype of the disease^42^, chronic kidney disease^42^, length of hospital stay^42^, smoking history^42^, forced vital capacity^42^, number of comorbidities^43^, acute respiratory distress syndrome class^43^, duration of invasive mechanical ventilation^43^ ^,^ ^55^, occupational status^43^, and intensive care requirement^35^ ^,^ ^54^, body mass index^55^, sleep apnea^55^, undergraduate education^54^, unemployment status^54^, presence of diabetes^54^, and heart failure diagnosis^54^. Taken together, the recognition of these factors can help identify patients with worse HRQoL and should be considered when managing patients to improve their HRQoL.

### FINAL CONSIDERATIONS

In summary, the present study suggests that, in post-COVID-19 patients who required hospitalization, (1) HRQoL partially improved soon after hospital discharge; (2) HRQoL impairment persists for months, both in physical and mental aspects; (3) the HRQoL in patients who were infected is worse when compared to uninfected individuals, even months after hospital discharge; (4) the HRQoL seems to be worse in patients admitted to the intensive care unit when compared to those who remained in the ward; (5) improvement in the HRQoL of patients after hospital discharge is independent of imaging improvement; (6) there is no evidence to support the association between HRQoL after hospital discharge and disease severity on hospital admission; (7) women and old age are the most established determinants of HRQoL; and (8) other clinical, demographic, and lifestyle factors may be associated with the HRQoL of patients and should be used to develop tailored strategies in their clinical management.
